# Elevated serum S14 levels are associated with more severe liver steatosis by ultrasonography

**DOI:** 10.1038/s41598-021-03279-8

**Published:** 2021-12-17

**Authors:** Wen-Ti Lin, Kuen-Cheh Yang, Yen-Ting Chen, Kuo-Chin Huang, Wei-Shiung Yang

**Affiliations:** 1 Graduate Institute of Clinical Medicine, College of Medicine, National Taiwan University, Taipei, Taiwan; 2 Department of Family and Community Medicine, Chung Shan Medical University Hospital, Taichung, Taiwan; 3 School of Medicine, Chung Shan Medical University, Taichung, Taiwan; 4 Department of Family Medicine, National Taiwan University Hospital Bei-Hu Branch, Taipei, Taiwan; 5 Department of Family Medicine, National Taiwan University Hospital, Taipei, Taiwan; 6 Department of Family Medicine, College of Medicine, National Taiwan University Hospital, Bei-Hu branch, No. 87, Neijiang St., Wanhua Dist., Taipei, Taiwan; 7 Division of Endocrinology and Metabolism, Department of Internal Medicine, National Taiwan University Hospital, No. 7, Chung-Shan South Road, Taipei, Taiwan

**Keywords:** Biomarkers, Endocrinology, Gastroenterology, Molecular medicine

## Abstract

S14 has been identified as a potent stimulator of de novo hepatic lipogenesis (DNL) in rodents. However, it is unclear how S14 is regulated in humans with non-alcoholic fatty liver disease (NAFLD). The aim of this study was to investigate the relationship between serum S14 and liver steatosis in humans with NAFLD. A total of 614 participants were recruited from community. Liver steatosis were evaluated according to the Ultrasonographic Fatty Liver Indicator (US-FLI), which is a semi-quantitative liver ultrasound score. Anthropometric and biochemical indices were collected for further analysis. The risk of liver steatosis severity was estimated by a cumulative logistic regression model. NAFLD was found in 52.2% of the participants. The subjects with NAFLD showed higher levels of waist circumference, body mass index, insulin resistance, aspartate aminotransferase, dyslipidemia, visceral fat, serum S14 and risk of metabolic syndrome (MetS) than those of controls. Compared with the first tertile of serum S14, the odds ratios for the risk of more severe liver steatosis were 1.22 (95% confidence interval [CI]: 0.78–1.92) for those of the second tertile and 2.08 (95% CI: 1.28–3.39) for the third tertile (P for trend < 0.05) after adjusting for confounding factors. Higher serum S14 level was not only found in NAFLD subjects but also was positively correlated with the severity of liver steatosis. S14 may play an important role in the mechanism of DNL for NAFLD in humans.

## Introduction

Non-alcoholic fatty liver disease (NAFLD), defined as excess triglycerides accumulation in the liver, has become the most common cause for chronic liver disease in developed countries and is estimated to impact at least 30% of Americans^1,2^ and 23–32% of Asian people^3^. Obesity, dyslipidemia and type 2 diabetes are well-known risk factors for the development of NAFLD. In subjects with NAFLD, the primary event is over-accumulation of triglycerides in hepatocytes. Donnelly et al.^4^ reported that fatty acid newly made within the hepatocytes through de novo lipogenesis (DNL) is one of the major sources of triglycerides in the liver. Other studies have also demonstrated that abnormally upregulated DNL is a distinct characteristic of individuals with NAFLD^4,5^. The full understanding of the mechanisms between NAFLD and DNL is of extreme importance and may provide new insight into identifying new targets for treatment of NAFLD.

Recently, S14 (spot 14) or termed thyroid hormone responsive spot 14 protein (THRSP), was reported to be a potent modulator of lipid synthesis. This gene is predominantly expressed in hepatic, adipose and lactating mammary tissues, and its expression has been found to respond rapidly to stimulation of thyroid hormones and carbohydrate^6–8^. Previous studies have shown that S14 plays a key role in the DNL process by modulating lipogenic genes such as SREBP-1c and LXR^9^. In animal models, overexpression of S14 in C57B2/6 mice promoted hepatic lipogenesis, whereas a decrease in S14 expression by siRNA (small interfering RNA) in db/db mice showed an opposite result^9^. In addition, S14 knockdown rendered marked inhibition of lipogenic enzyme immunoreactivities in primary culture of rat hepatocytes^10^. Furthermore, the S14 null mice exhibited resistance to diet-induced obesity, improved glucose tolerance and insulin sensitivity^11^. Human NAFLD data regarding S14 are relatively lacking and focused mainly on obesity, not NAFLD. In studies of obese human, S14 mRNA level in adipose tissue was abnormally regulated^12^ and was lower in adipose tissue of obese subjects^13^, which seemed to be inconsistent with the observation of the S14 null mice experiment^11^. Further studies are needed to elucidate this controversy issue.

Since S14 plays an important role in lipogenesis, we speculated that S14 may participate in the NAFLD development. In fact, novel anti-NAFLD treatment targeting on DNL is in development^14^. Zeng et al. reported that MiR-451a represented a new potential target for NAFLD through regulating S14 gene expression^15^. Further research is needed to clarify the contribution of S14 to the development of NAFLD. Therefore, the objective of this study was to investigate the relationship between serum S14 concentrations and the severity of liver steatosis in NAFLD subjects.

## Methods

### Subjects and data collection

We recruited 614 healthy volunteers in the community of Hsinchu city, Taiwan. The study protocol was approved by the Institutional Review Board of National Taiwan University Hospital (IRB NO. 201210012RIC) and was carried out in accordance with its guideline. All the subjects provided written informed consent and the details were described in our previous study^16^. All volunteers did not have excess alcohol intake (> 30 g and > 20 g for men and women, respectively) or chronic liver diseases^16^. Completed detailed surveys that included standardized questionnaires and the evaluation of the medical and personal history were administered by our examiners.

### Anthropometric and laboratory measurements

Anthropometric data were collected, and biochemical analyses were performed in routine health examinations. We calculated body mass index (BMI) as weight/height^2^ (kg/m^2^). Standard body mass index (BMI) was calculated and the cutoffs were defined according to the Department of Health in Taiwan: optimal BMI was defined as 18.5 ≤ BMI < 24 kg/m^2^ (non-obese group), overweight and obesity were defined as BMI ≥ 24 kg/m^2^ (overweight and obese group). Waist circumference was measured at the midpoint between the lower border of the rib cage and iliac crest. Blood samples were drawn from each participant after 12 h of overnight fasting and were immediately stored at − 80 °C for. The homeostasis model assessment estimate of insulin resistance (HOMA-IR) was calculated from plasma insulin and glucose values, which were developed by the Diabetes Trials Unit of the Oxford Center for Diabetes, Endocrinology, and Metabolism (https://www.dtu.ox.ac.uk/homacalculator). Trunk fat percentage (0–75%) was measured by bioelectrical impedance analysis (BIA) using Tanita AB-140 Viscan (Tanita Corp, Tokyo, Japan). The trunk fat percentage are significantly with total abdominal adipose tissue^17^ and total subcutaneous abdominal adipose tissue^18^. Body fat and visceral fat were measured by BIA using Tanita MC-980 (Tanita Corp, Tokyo, Japan) and shown by percentage and visceral fat rating (VFR, score ranges from 1–59). A rating between 1 and 12 indicates healthy level and a 13–59 indicates excessive level of visceral fat.

### S14 immunoassay

We developed an ELISA to measure serum S14 levels in our lab as previously described^19^. In brief, polystyrene MaxiSorp 96-well plates (Nunc A/S, Roskilde, Denmark) were coated with 100 μL/well human recombinant S14 proteins (100 ng/mL, diluted in PBS; cat no. ag3721, ProteinTech, Chicago, IL). The coated plates were sealed and incubated on an orbital shaker (at 100 rpm; OS701, KS, Taiwan) at 4 °C overnight. The liquid was removed and the plates were washed in washing buffer (PBS-Tween (PBS-T), 0.05% Tween 20), and were pad-dried on paper towel. The plates were blocked with 100 μL blocking buffer/well (PBS-T with 1% BSA), and incubated at 4 °C overnight at 100 rpm on the orbital shaker. After washing with PBS-T three times and dried, 50 μL serum samples were added into each well and incubated for 1 h at room temperature (RT) on a rotor at 150 rpm. Subsequently, rabbit anti-S14 polyclonal antibody (diluted in blocking buffer by 1:10,000, catalog no. 13054-1-AP, ProteinTech, Chicago, IL) were added (50 μL per well) and incubated for 2 h at RT shaken at 150 rpm. After washing with PBS-T three times, horseradish peroxidase-conjugated goat anti-rabbit IgG polyclonal antibody (diluted in blocking buffer by 1:10,000; GTX213110-01, Irvine, CA) was added (100 μL per well) and shaken (150 rpm) for 1 h at RT. Following five times of washing with PBS-T, color was developed using the 100 μL 3,3′,5,5′-tetramethylbenzidine (TMB) solution (catalog no. 53-00-03,KPL, Gaithersburg, MD) each well. After 10-min incubation, the reaction was stopped by adding 100 μL 2.0 M H_2_SO_4_ per well. The absorbance was measured immediately at 450 nm by microplate reader (VERSA max, Munich, Germany). Four-parameter logistic model was used to draw the standard curve. For the sensitivity, the minimum detection limit was 10 ng/mL. For the intra-assay variability, the coefficient of variance (CV) of 6 replicate sets of one serum sample was 7.5%. For the inter-assay variability, the CV of 6 independent assays of one serum sample was 9.5%.

### Abdominal ultrasonography for NAFLD

Hepatic ultrasonography was performed in all participants, after an 8-h overnight fasting by well-trained physicians with a 3.5–5 MHz transducer and a high-resolution ultrasonographic system (Hitachi Aloka ProSound α6). The severity of hepatic steatosis was quantified by Ultrasonographic Fatty Liver Indicator (US-FLI)^20^, a semi-quantitative scoring system, which ranges from 0 to 8. Before the study, all of the physicians reached a consensus concerning the standard procedure for ultrasound scanning^16^. A similar study of US-FLI has demonstrated a good inter-observer agreement (κ = 0.805–0.882, P < 0.001)^21^. The semi-quantitative US-FLI is composed of five indicators: (1) presence of liver-kidney contrast graded as mild/moderate (score 2) to severe (score 3); and (2) presence (score 1) or absence (score 0) of posterior attenuation of the ultrasound beam, vessel blurring, difficult visualization of the gallbladder wall, difficult visualization of the diaphragm and areas of focal sparing (each score 1). Subjects were divided into three groups according to the severity of ultrasonographic liver steatosis by the US-FLI score: normal (score 0–1), mild steatosis (score 2–4), and moderate-to-severe steatosis (score ≥ 5). Consistency of various severity of liver steatosis between US-FLI and histological findings was demonstrated to be good^21^.

### The definition of metabolic syndrome

The diagnosis of metabolic syndrome (MetS) was based on the modified National Cholesterol Education Program Adult Treatment Panel III Criteria (NCEP-ATP III) for the Asian population. MetS was present if three or more of the following five criteria were met: (1) a WC of ≥ 90 cm for men or ≥ 80 cm for women; (2) a systolic blood pressure ≥ 130 mmHg, or a diastolic blood pressure ≥ 85 mmHg, or the use of medications for hypertension; (3) hyperglycemia (FPG ≥ 100 mg/dL) or the use of medications for diabetes; (4) hypertriglyceridemia (TG ≥ 150 mg/dL) or the use of medications for hyperlipidemia; and (5) low HDL-C (≤ 40 mg/dL in men and ≤ 50 mg/dL in women) or the use of medications for hyperlipidemia.

### Statistical analysis

Data are expressed as mean ± standard deviation (SD) and as percentages respectively for continuous and categorical variables. Because of skewed distributions, the natural logarithmic (ln) transformations were performed for S14 values to approximate a normal distribution before analysis. Difference between groups were tested using an independent t-test for continuous variables, and the Pearson Chi-squared test for categorical variables. The relationships between serum S14 level and metabolic factors were explored by Pearson correlation coefficient (r).

The risk of liver steatosis severity for serum S14 level was shown by odds ratios (OR) and 95% confidence interval (CI) using a cumulative logistic regression model. It was performed by using liver steatosis severity as a dependent variable (normal, mild and moderate-to-severe) and S14 tertile as an independent variable with adjustment for age, sex, HOMA-IR, MetS, CRP, menopause, exercise time, and smoking status. The ranges of the first, second and third tertiles of S14 were 11.98–71.8, 71.9–115.1, 115.2–588.3 ng/mL, respectively. The least square means (LSMs) of serum S14 level was calculated and compared in the generalized linear model after adjustment for above confounding factors. In order to clarify the effect of visceral fat, we further analyzed the LSMs of S14 in mild and moderate to severe NAFLD subjects stratified by three VFR group [high VFR group (score 10–25), medium VFR group (score 6–9), low VFR group (score 1–5)]. All analyses were performed with SPSS 20.0. A P value of less than 0.05 indicated statistical significance.

## Results

Six participants were excluded because their serum S14 were outlier. From 608 participants, 327 (52.2%) cases had NAFLD and among them, 220 (35.5%) and 107 (16.7%) were classified as mild and moderate-to-severe, respectively. The clinical and metabolic characteristics of the participants are summarized in Table 1. Waist circumference, BMI, insulin, HOMA-IR (homeostasis Model Assessment for Insulin Resistance), ALT (Alanine aminotransferase), triglycerides, lower HDL-C (high-density lipoprotein (HDL) cholesterol), trunk fat percentage, VFR (visceral fat rating), MetS (metabolic syndrome) were comparatively more abnormal or prevalent in NAFLD patients than control group in both non-obese (BMI < 24) and overweight/obese group (BMI ≥ 24). In the non-obese group, the serum S14 was 108.5 ± 45.4 ng/dL and 96.3 ± 44.4 ng/dL respectively in subjects with and without NAFLD (P = 0.023). Similarly, in the overweight/obese group, the serum S14 was 96.4 ± 45.6 ng/dL and 84.2 ± 41.6 ng/dL respectively in subjects with and without NAFLD (P = 0.06).

**Table 1:** Baseline characteristics and clinical variables stratified by the presence of NAFLD and BMI level.

Variable	All (N = 608)	Non-obese group (BMI < 24)			Overweight and obese group (BMI ≥ 24)		
		Non-NAFLD (N = 226)	NAFLD (N = 107)	P-value	Non-NAFLD (N = 55)	NAFLD (N = 220)	P-value
Male (%)	236 (38.8)	48 (21.2)	38 (35.5)	0.005	26 (47.3)	124 (56.4)	0.226
Age (years)	42.7 ± 11.5	41.1 ± 11.0	42.9 ± 11.9	0.185	44.8 ± 11.4	43.7 ± 11.7	0.512
Waist (cm)	81.1 ± 10.7	73.0 ± 6.2	77.6 ± 6.5	0.000	85.4 ± 6.2	91.0 ± 8.3	0.000
BMI (kg/m^2^)	24.0 ± 4.4	20.6 ± 1.9	21.9 ± 1.5	0.000	26.0 ± 1.7	28.2 ± 3.9	0.000
FPG (mg/dL)	88.1 ± 17.3	83.8 ± 13.0	85.2 ± 8.6	0.244	87.0 ± 10.3	94.3 ± 23.0	0.021
Insulin (μU/mL)	8.3 ± 7.2	5.3 ± 4.3	6.8 ± 5.2	0.021	7.0 ± 3.8	11.5 ± 8.9	0.001
HOMA-IR	1.07 ± 0.9	0.68 ± 0.6	0.86 ± 0.65	0.022	0.9 ± 0.5	1.5 ± 1.1	0.001
AST (U/L)	22.7 ± 8.5	20.4 ± 6.8	21.8 ± 7.0	0.080	21.6 ± 10.6	36.5 ± 27.7	0.000
ALT (U/L)	25.8 ± 21.0	17.2 ± 9.4	24.1 ± 16.4	0.000	21.7 ± 6.0	25.8 ± 10.2	0.000
TCHO (mg/dL)	196.1 ± 35.4	190.4 ± 33.7	197.8 ± 39.9	0.076	194.4 ± 29.1	201.5 ± 35.7	0.174
Triglycerides (mg/dL)	113.8 ± 88.3	74.4 ± 37.2	110.2 ± 78.9	0.000	96.0 ± 43.7	160.6 ± 113.3	0.000
HDL-C (mg/dL)	58.3 ± 15.6	66.8 ± 15.1	57.2 ± 13.2	0.000	59.6 ± 13.4	49.8 ± 12.7	0.000
LDL-C (mg/dL)	123.6 ± 33.3	114.7 ± 31.1	126.4 ± 37.1	0.003	122.7 ± 29.0	131.5 ± 32.5	0.052
Creatinine (mg/dL)	0.84 ± 0.18	0.78 ± 0.16	0.82 ± 0.17	0.036	0.89 ± 0.20	0.91 ± 0.18	0.554
**US-FLI score**	2.1 ± 2.2	0.1 ± 0.3	2.7 ± 1.1	0.000	0.2 ± 0.4	4.2 ± 1.7	0.000
Mild (%)	327 (53.8)	–	–	–	–	–	–
Moderate-and-severe (%)	105 (17.3)	–	–	–	–	–	–
Body fat (%)	28.5 ± 7.9	25.4 ± 6.3	26.5 ± 6.0	0.122	29.9 ± 7.8	32.5 ± 8.4	0.038
Truncal fat	31.7 ± 8.7	27.3 ± 8.1	30.7 ± 7.3	0.000	33.9 ± 7.0	36.3 ± 7.9	0.027
VFR	7.8 ± 4.5	4.4 ± 2.5	6.1 ± 2.7	0.000	9.5 ± 2.7	11.8 ± 3.6	0.000
Metabolic syndrome (%)	108 (17.8)	4 (1.8)	9 (8.4)	0.003	5 (0.1)	90 (40.9)	0.000
S14 (ng/mL)	97.4 ± 45.1	96.3 ± 44.4	108.5 ± 45.4	0.023	84.2 ± 41.6	96.4 ± 45.6	0.06

*BMI* body mass index, *FPG* fasting plasma glucose, *HOMA-IR* homeostasis model assessment of insulin resistance, *AST* aspartate aminotransferase, *ALT* alanine aminotransferase, *TCHO* total cholesterol, *HDL* high-density lipoprotein, *LDL* low-density lipoprotein, *US-FLI* Ultrasonography Fatty Liver Index (score: 0–8), *VFR* visceral fat rating (score: 1–59).

Table 2 showed the Pearson correlation coefficients between serum S14 and metabolic factors. There were significant negative correlations between serum S14 level and age (*ρ* = − 0.359, P = 0.001), waist circumference (*ρ* = − 0.104, P = 0.014), fasting plasma glucose (*ρ* = − 0.087, P = 0.031), total cholesterol (*ρ* = − 0.107, P = 0.009), serum triglycerides (*ρ* = − 0.107, P = 0.008), trunk fat percentage (*ρ* = − 0.141, P = 0.001) and VFR (*ρ* = − 0.108, P = 0.008). Nevertheless, no significant statistical correlation was observed between S14 level and HOMA-IR and body fat percentage.

**Table 2:** Correlation coefficients between S14 and anthropometric and metabolic factors.

Impendent variables	All (male and female, N = 608)	
	R	P
Age (years)	− 0.359	0.000**
Waist (cm)	− 0.104	0.014*
Body weight (kg)	− 0.021	0.613
BMI (kg/m^2^)	− 0.044	0.281
Creatinine (mg/dL)	0.002	0.966
FPG (mg/dL)	− 0.087	0.031*
Insulin (μU/mL)	0.007	0.874
HOMA-IR	0.007	0.866
AST (U/L)	− 0.017	0.672
ALT (U/L)	0.032	0.432
TCHO (mg/dL)	− 0.107	0.009*
Triglycerides (mg/dL)	− 0.107	0.008*
HDL-C (mg/dL)	0.042	0.305
LDL-C (mg/dL)	− 0.066	0.103
Body fat (%)	− 0.035	0.395
Truncal fat (%)	− 0.141	0.001**
VFR	− 0.108	0.008*

*BMI* body mass index, *FPG* fasting plasma glucose, *HOMA-IR* homeostasis model assessment of insulin resistance, *AST* aspartate aminotransferase, *ALT* alanine aminotransferase, *TCHO* total cholesterol, *HDL* high-density lipoprotein, *LDL* low-density lipoprotein, *US-FLI* Ultrasonography Fatty Liver Index (score: 0–8), Mild (≥ 2), Moderate-and-severe (> 4), *VFR* visceral fat rating.

We performed cumulative logistic regression analysis to examine the relationship between the severity of liver steatosis with serum S14 (Table 3). In model 1, adjusted for age, sex and BMI, the subjects in the highest S14 tertile showed a higher risk for NAFLD compared to those in the lowest S14 tertile (OR 1.86; 95% CI 1.21–2.87; P for trend < 0.05). In the models 2 and 3 after further adjustment for a series of confounders such as HOMA-IR, CRP, metabolic syndrome, smoking behavior, exercise time and menopause, there was still a dose response for higher risk of NAFLD severity with higher level of S14 (P for trend < 0.05). The ORs for more severe NAFLD were 1.22 (95% CI 0.78–1.92) and 2.08 (95% CI 1.28–3.39) respectively for the second tertile and the highest tertile of S14 as compared to the lowest tertile (Table 3).

**Table 3:** The cumulative risk for more severe non-alcoholic fatty liver disease of serum S14 (N = 608).

Tertile of S14^†^	Model 1		Model 2		Model 3	
	OR (95% CI)	P for trend	OR (95% CI)	P for trend	OR (95% CI)	P for trend
Tertile 1	Reference	< 0.05	Reference	< 0.05	Reference	< 0.05
Tertile 2	1.13 (1.34–1.71)		1.18 (0.76–1.84)		1.22 (0.78–1.92)	
Tertile 3	1.86 (1.21–2.87)		1.98 (1.23–3.19)		2.08 (1.28–3.39)	

Model 1: adjusted for age, sex and obesity (OR = 8.88 [6.14–12.83], P < 0.0001). Obesity is defined by BMI ≥ 27.

Model 2: model 1 plus HOMA-IR (OR: 1.47 [1.15–1.88], P = 0.002), CRP, metabolic syndrome (OR: 4.08 [2.46–6.75], P < 0.0001).

Model 3: model 2 plus smoking behavior, exercise time per week and menopause (women only).

^†^S14 was transformed by nature logarithm; the severity of NAFLD was classified by normal, mild, and moderate-to-severe.Ranges of S14 tertile: first tertile (11.98–71.8), second tertile (71.9–115.1), third tertile (115.2–588.3).

**Figure 1:**
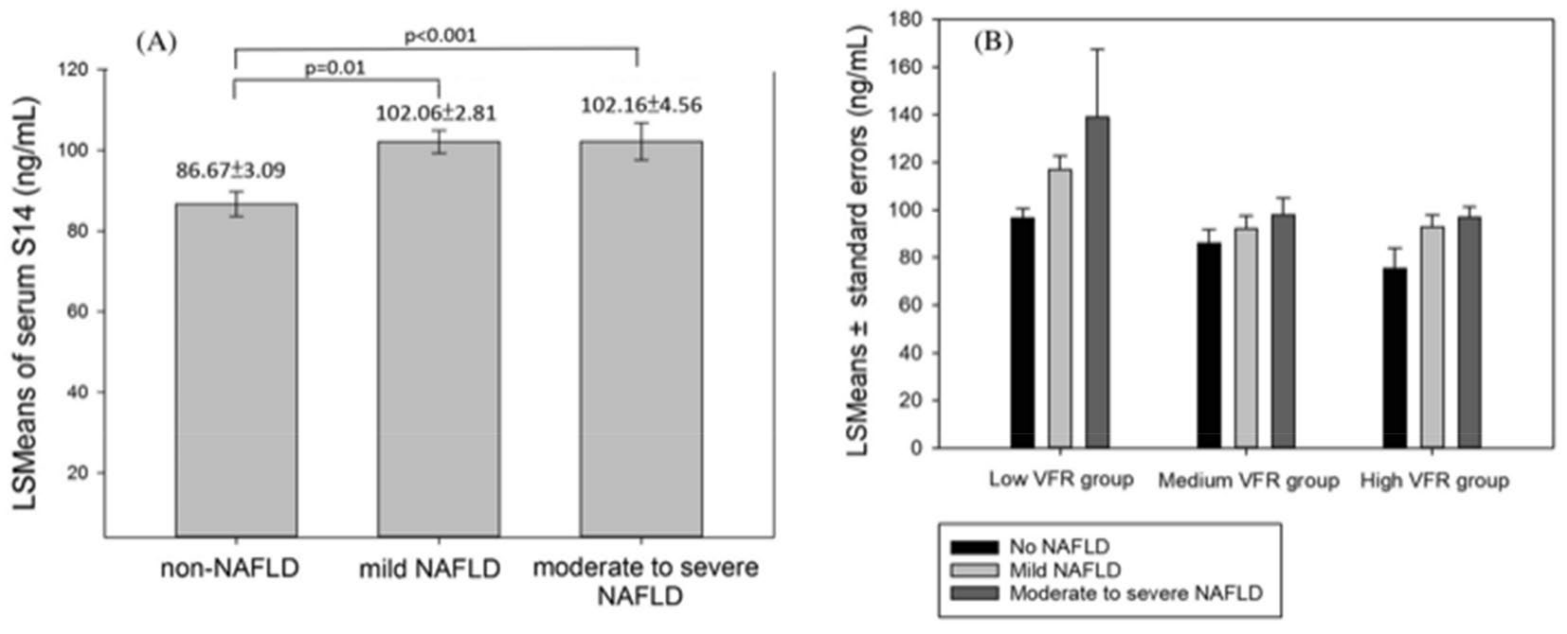
The least square (LS) means of (**A**) serum S14 level adjusted for age, sex, obesity, HOMA-IR, CRP, metabolic syndrome, smoking behavior, exercise time and menopause by the status of NAFLD and (**B**) serum S14 level adjusted for age, sex, BMI, HOMA-IR, CRP, metabolic syndrome, smoking behavior, exercise time and menopause stratified by visceral fat rating. *VFR visceral fat rating (score: 1–25), High (10–25), Medium (6–9), Low VFR (1–5).

The least square means of serum S14 level in mild and moderate to severe NAFLD groups after adjusting confounding factors (age, sex, obesity, HOMA-IR, CRP, metabolic syndrome, exercise time, menopause, smoking behavior) were significantly higher than the control group (adjusted means ± SE: 102.06 ± 2.81, 102.16 ± 4.56 vs. 86.67 ± 3.09; 95% CI 95.53–107.59, 93.21–111.11 vs. 80.60–92.74, respectively. P < 0.0001) in Fig. 1A.

The least square means of serum S14 level were estimated after adjustment for potential confounding factors (age, sex, BMI, HOMA-IR, CRP, metabolic syndrome, exercise time, menopause, smoking behavior) with different visceral fat level stratification (Fig. 1B). The serum S14 levels were lower in the subjects of higher visceral fat. However, we found that serum S14 level remained significant higher in more severe NAFLD (P < 0.05) across all VFR groups.

## Discussion

In our study, we demonstrated that with increasing severity of liver steatosis, there were statistically significant increase in serum S14 levels after considering the insulin resistance, BMI and metabolic syndrome. To the best of our knowledge, this is the first study to investigate the relationship between serum S14 levels and NAFLD in human subjects.

NAFLD is now the leading cause of liver disease in developed countries. Despite its high prevalence, knowledge on the pathogenesis of NAFLD was still incomplete. The widely accepted “multiple hit theory” provides the explanation for NAFLD development. It is postulated that the primary event is over-accumulation of triglycerides in hepatocytes^22^. According to previous studies, the contribution of DNL to the hepatic total TG content in the fasting status was very small (less than 5%) and elevated following meals (23%) in healthy human subjects^23^. In contrast, DNL activity in subjects with NAFLD is already elevated in the fasting status (26% ± 7%)^4^. Accordingly, enhanced liver DNL appears to be one of the major abnormalities of hepatic fat metabolism in subjects with NAFLD.

S14 has been reported to closely link to DNL. It is a 17-kDa nuclear protein mainly expressed in lipogenic tissues and is postulated to transduce hormone-related or nutrient-related signals to lipogenic genes through a molecular mechanism not yet elucidated. *S14* gene may act as a key lipogenic transcriptional cofactor^24^ and is induced rapidly by thyroid hormone, carbohydrate intake, adipose tissue differentiation, insulin and lactation^6,25^. Studies have shown that S14 is important for the biosynthesis of triglycerides with medium-length fatty acid chain and is regulated through interactions with lipogenic factors such as thyroid receptor, ACC (acetyl‐CoA carboxylase)^26^, SREBP-1c^27^, PXR (the pregnane X receptor)^28^, LXR (liver X receptor)^9^ and CAR (constitutive androstane receptor)^29^. In animal model, rat hepatocytes with S14 knockdown showed marked reduction of triglycerides formation^10^. Overexpression of S14 led to increased triglyceride accumulation via enhanced lipogenic genes expression (SREBP-1c, FAS, DGAT) in livers of C57Bl/6 mice^9^. In our study, higher S14 level predicted more severe NAFLD after adjusting potential confounding factors (Table 3), our data implied that DNL was abnormally upregulated in NAFLD subjects, which is consistent with previous studies^4,5^.

The serum S14 was negatively linked to visceral fat. However, BMI and total body fat percentage showed negative associations with serum S14 without statistical significance, which implied that S14 may be related to abdominal adiposity than general obesity. Similar to our findings, Ortega et al. showed that S14 mRNA level in abdominal omental adipose tissue was negatively associated with BMI and percentage of fat mass^13^. Kirschner et al. also found that S14 gene expression level was strongly down-regulated in the abdominal adipose tissue of non-obese subjects in response to fasting, but only minimally down-regulated in obese individuals^12^. Our prior study and this study^30^ both supported that patients with metabolic syndrome had lower serum S14 level than those without.

Previous studies exploring lipogenic genes expression in obese subjects may partly explain this contradictory finding between lower serum S14 level and cardiometabolic factors. After a large and long-lasting fat excess, the decreased expression level of lipogenic genes could be a late and adaptive process, aiming at limiting adipocyte hypertrophy and further development of fat mass, including liver fat accumulation. The supportive evidence is that a remarkable reduction in the expression of genes coding for lipogenic factors such as SREBP-1c, FASN, ACC, PEPCK, ATP Citratelyase, or Pyruvate Carboxylase^31–33^ or involved in adipocyte differentiation^34^ has been found in obese subjects. As S14 regulates lipogenesis partly via interaction with lipogenic factors, we speculate that, as the fat mass increases and the cardiometabolic risk factors develop, downregulation of S14 in adipose tissue initiates to limit its expansion. Although NAFLD and some of the cardiometabolic factors share similar pathophysiology, there is no direct evidence whether this negative feedback of lipogenic factors also occurs in NAFLD as cardiometabolic factors do. In our study, we observed that serum S14 level in subjects with NAFLD remained high and was positively associated with NAFLD severity. Additionally, subjects with NAFLD had significantly higher serum S14 levels despite their visceral fat severity (Fig. 1B).

Interestingly, the serum S14 was negatively linked to cardiometabolic factors including increased age, waist circumference, fasting plasma glucose, serum total cholesterol, triglycerides and visceral fat, all of which were reported risk factors of NAFLD. This paradox in our results was the apparent selective nature of S14—wherein S14 seemed to be negatively associated with abnormal cardiometabolic factors yet correlated positively with hepatic steatosis. One of the explanations may be that the negative feedback between S14 and excess liver fat was impaired in NAFLD subjects.

Our study has several limitations to be considered. First, it was a cross sectional study and could not determine the causality. Second, we did not have thyroid hormone data since it served as a confounding factor of S14 level although we had the past history of thyroid status. Third, we did not perform liver biopsies for the diagnosis of NAFLD. Although liver biopsy is regarded as a gold standard for NAFLD diagnosis, it is invasive and associated with morbidities and mortality. Fourth, our ELISA kit used a polyclonal antibody against S14, it may detect S14-R as well since the S14R protein is 32% homologous to S14 in amino acid sequences. Specific monoclonal antibodies should be generated in the future to solve this problem. Lastly, the exact contribution of serum S14 from different organs in human remains unknown. Further studies are required to elucidate this issue. Advantages of the present study included relatively large sample size and the participants were recruited from the community, thus can be applied to the general population. In addition, we used US-FLI score, which is known to have good correlation with liver CT and histology providing the quantitative information of liver fat^21^.

In conclusion, we showed that serum S14 level increased with the severity of hepatic steatosis. However, the serum level was inversely associated with metabolic factors, which probably come from the negative feedback or rescue response of S14. The detailed mechanisms of S14 protein involved in DNL needs further research in the future.
